# The *ATRX* splicing variant c.21-1G>A is asymptomatic

**DOI:** 10.1038/s41439-022-00212-x

**Published:** 2022-09-14

**Authors:** Karin Kojima, Takahito Wada, Hiroko Shimbo, Takahiro Ikeda, Eriko F. Jimbo, Hirotomo Saitsu, Naomichi Matsumoto, Takanori Yamagata

**Affiliations:** 1 Department of Pediatrics, Jichi Medical University, 3311-1 Yakushiji, Shimotsuke-shi, Tochigi 329-0498 Japan; 2 Department of Genomic Medicine, Kyoto University Graduate School of Medicine, Oshida-Konoe-cho, Sakyo-ku, Kyoto, 606-8501 Japan; 3 Department of Neurology, Kanagawa Children’s Medical Center, 2-138-4 Mutsukawa, Minami-ku, Yokohama, Kanagawa 232-8555 Japan; 4 Department of Human Genetics, Yokohama City University Graduate School of Medicine, 3-9 Fukuura, Kanazawa-ku, Yokohama, Kanagawa 236-0004 Japan

**Keywords:** Disease genetics, Genetic counselling

## Abstract

The *ATRX* variant c.21-1G>A was detected by an exome analysis of a patient with Cockayne syndrome without alpha thalassemia X-linked intellectual disability syndrome (ATR-XS). In addition, variants in *ERCC6* were detected. *ATRX* c.21-1G>A is localized at the splicing acceptor site of intron 1. This splicing event, NM_000489.6: c.21_133del p.S7Rfs*1, induces exon 2 deletion and early termination. The start codon in exon 3 of *ATRX* is presumed to produce a slightly shorter but functional ATRX protein.

*ATRX* at Xq13.3 is a causative gene for alpha thalassemia X-linked intellectual disability syndrome (ATR-XS)^1^. *ATRX* has 35 exons. ATR-XS is a rare genetic disorder affecting multiple organ systems that present with intellectual disability (ID), characteristic facial features, microcephaly, genital abnormalities, bone abnormalities, and alpha thalassemia^1^. Characteristic facial features include an upward-turned nose, thick lower lip, low nasal bridge, triangle mouse, incisors with gap, and low-set ears. Genital abnormalities include micropenis, micro/retention of testicles, and hypospadias. Bone abnormalities include tapering fingers, shortening of fifth fingers, and contracture of finger joints. Thus far, many missense and nonsense variants throughout the entire gene have been reported, and variants in the PHD-like domain have been reported to be related to a severe phenotype^2^.

Cockayne syndrome (CS) is also a rare autosomal recessive disorder that presents with cachectic dwarfism, severe ID, progeroid appearance, sensorineural hearing loss, and intracranial calcification^3^. Variants in *ERCC6* at 10q11.23^4,5^ are the predominant cause of CS, and variants in other genes, such as *ERCC8*^6^, *ERCC5*^7^, *ERCC3*^8^, and *ERCC4*^7^, have been reported.

Using exome analysis for a male CS patient, we identified *ERCC6* variants as well as an *ATRX* variant at the splice acceptor site of intron 1, even though this patient was not considered to have ATR-X syndrome.

A 12-year-old male born to nonconsanguineous parents was delivered uneventfully. His birth weight was 3.36 kg (0.2 SD), height was 50 cm and head circumference was 34.5 cm. He had shown head control at 5 months, standing with support at 11 months, and standing independently at 2 years and 10 months old. He was introduced to our hospital at 5 years old. At this time, his height was 91 cm (−4.3 standard deviations [SD]), his weight was 14.7 kg (−1.4 SD), and head circumference was 46.7 cm (−3.1 SD). He had severe ID and could speak only three simple words, although he could understand simple verbal orders. He had spastic diplegia with knee and ankle joint contracture and could walk several steps. He did not have any genital abnormalities, as he did not present with either micropenis or cryptorchidism. Additionally, he did not have finger abnormalities. Cranial CT showed calcification of the bilateral globus pallidus. Magnetic resonance imaging (MRI) showed mild cerebral and cerebellar atrophy, with T2 high intensity in the white matter mainly in the bilateral frontal to parietal areas. His blood analysis data were as follows: RBC 422 × 10^4^/μL, Hb 11.9 g/dL, Ht 34.6%, plt 26.2 × 10^4^/μL, AST 51 U/L, ALT 54 U/L and CPK 109 U/L. Alpha thalassemia was not detected. Hemoglobin H inclusion bodies in red blood cells were not detected by brilliant cresyl blue staining (data not shown). His facial appearance of a bird-like/progeroid appearance, including the features of hollow eyes and cheeks and a beak-like nose, gradually became evident. However, characteristic facial features of ATR-XS were not observed.

Lymphocytes and fibroblasts were obtained from the patient, and lymphocytes were obtained from his mother. Samples from the father were not available. The mother gave her informed consent for the patient herself to undergo analyses. Informed consent acquisition and genetic analyses were performed in accordance with the human study protocols approved by the institutional review board at Kanagawa Children’s Medical Centre, Yokohama City University School of Medicine and Jichi Medical University.

Genomic DNA was captured using the SureSelect Human All Exon v4 Kit (51 Mb; Agilent Technologies, Santa Clara, CA, USA). It was sequenced with an Illumina HiSeq2000 apparatus (Illumina, San Diego, CA, USA), which generated 101-bp paired-end reads. Exome data processing was performed according to the best practice of the Genome Analysis Toolkit (version 2)^9^.

Potential pathologic variants were confirmed by Sanger sequencing using an ABI 3500xl apparatus (Life Technologies, Carlsbad, CA, USA). Sequencing data were analyzed using a Sequencer (Gene Codes Corporation, Ann Arbor, MI, USA) with the primer set F;　CACCCACAACTGTAACATTTCC, and R; GCACATTCTTTTTCAATTTACCTG. RNA was extracted from lymphoblasts, and cDNA was synthesized using oligo dT. Reverse transcription polymerase chain reaction (RT‒PCR) was performed using a forward primer on exon 1 (GTTCCAGCAGCAGCTACAGTGAC) and a reverse primer on exons 3/4 (GAAGAGCTAGTTCCCTCTTCCTTGC) to detect possible changes in exon 2 splicing (Fig. 2a).

Immunostaining of the patient’s fibroblasts using a rabbit polyclonal antibody (H-300, Santa Cruz Biotechnology, Dallas, Texas) for the C-terminal region (amino acids 2193–2492) of ATRX detected the expression of ATRX in nuclei (Fig. 2g, h).

Protein was extracted from the patient’s lymphoblasts. The primary antibodies were rabbit anti-ATRX (ab97508) for the C-terminal region (amino acids 2211–2413) from Abcam (Eugene, OR, USA) and mouse anti-beta-actin from Sigma‒Aldrich (St. Louis, MO, USA). The membranes were incubated with horseradish peroxidase-conjugated secondary antibody (Santa Cruz, TX, USA) diluted in 5% phosphate-buffered saline. The secondary antibody was detected using the chemiluminescence ECL Plus reagent (Takara Bio, Shiga, Japan), and the membranes were visualized using the Image Quant LAS4000 Imaging System (GE Healthcare, Chicago, IL, USA).

In this patient, we detected three variants in *ERCC6*, NM_000124.4: c.1931T>C p. (Ile644Thr), c.1936del p. (Asp646Thrfs*52), and c.1627A>T p. (Ile543Phe), and one *ATRX* variant, NM_000489.6:c21-1G>A, at the splice acceptor site of intron 1—by whole-exome sequencing (Fig. 1a, b). All base changes were confirmed by direct sequencing (supplementary data). Variants c.1931T>C and c1936del in *ERCC6* and c21-1G>A in *ATRX* were also detected in the mother (Fig. 1a). According to the ACMG guidelines in 2015, the *ATRX* variant (NM_000489.6: 21-1G>A) was classified as “likely pathogenic” (PM1, PM2). Two *ERCC6* variants, NM_000124.4: c.1931T>C p. Ile644Thr and c.1627A>T p. Ile543Phe, were classified as “likely pathogenic”. Another *ERCC6* variant (NM_000124.4: c.1936del p. (Asp646Thrfs*52)) was classified as “uncertain significance”.

**Fig. 1:**
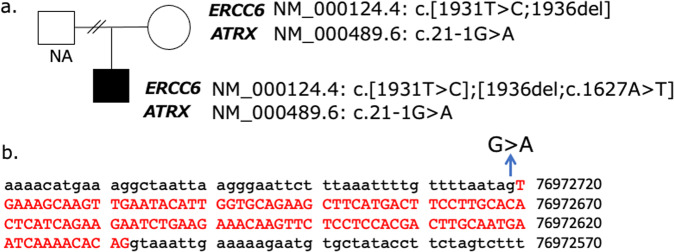
*ERCC6* and *ATRX* variants in the patient and his family. The pedigree of the patient is indicated in **a**. The patient had the c.1931T>C, c.1936del and c.1627A>T variants in *ERCC6*, and the c.21-1G>A variant in *ATRX*. His mother had the c.1931T>C and c.1936del variants in *ERCC6* and the c.21-1G>A variant in *ATRX*. His father’s sample was not available (NA). The position of c.21-1G>A in *ATRX* is indicated in **b**.

Because *ATRX* (c21-1 G>A) is a splice site variant (Fig. 1b), we analyzed the changes in exon 2 splicing by RT‒PCR using primer sets in exons 1 and 3–4 (Fig. 2a). The RT‒PCR product from the patient was approximately 100 bp shorter than that of the control (Fig. 2b). The sequence of this product detected exon 2 skipping, where the exon 3 sequence was observed after the exon 1 sequence without the exon 2 sequence (113 bp) (Fig. 2c). Due to this exon 2 skipping, c.21_133del, p.S7Rfs*1, an out-of-frame deletion, was induced. The seventh amino acid from the start codon (Ser) was changed to Arg, subsequently inducing a stop codon (Fig. 2d, e). In an immunoblot analysis using a rabbit polyclonal antibody that labels the C-terminus of ATRX, both the normal control and patient fibroblasts expressed ATRX in the nucleus (Fig. 2g, h), and the expression of ATRX was detected in patient lymphoblasts by Western blot analysis (Fig. 2i).

**Fig. 2:**
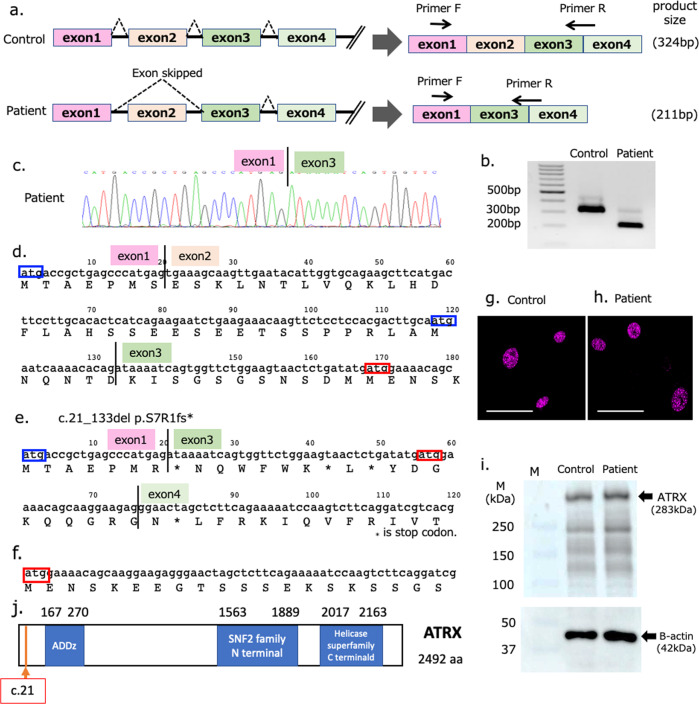
RT‒PCR and protein expression analyses of mutated *ATRX* mRNA. RT‒PCR was performed using a forward primer in exon 1 and a reverse primer spanning exons 3 and 4 (**a**). The RT‒PCR product of the patient was approximately 100 bp shorter than the control product (**b**), and a direct sequencing analysis confirmed exon 2 deletion. This sequence was spliced from exon 1 to 3, c.21_133del p.S7Rfs*1 (**c**, **d**, **e**). ATRX protein was expressed in the nuclei of the control fibroblasts (**g**) and in the patient’s (**h**) immunostained fibroblasts (scale bar: 50 μm). ATRX protein was also detected in the control and patient lymphoblasts (**i**). Start codons (ATG) were localized in exon 1 (blue square), exon 2 (blue square) and exon 3 (red square) (**d**). The start codon in exon 3 (red square) was considered to be active in the patient’s mutated mRNA to translate the ATRX protein (**f**). The position of c.21-1G>A in *ATRX* is located at the 7th amino acid in ATRX (**j**).

According to GenBank, three types of ATRX putative protein-coding sequences (CDSs) exist; these types have alternative start codons (ATG) in exon 1 (XP_005262214.2, etc.), exon 2 (XP_016885099.1, etc.) and exon 3 (XP_016885097.1, etc.) (Fig. 2d). In our patient, the CDS starting from exon 1 was out of frame after the seventh amino acid, resulting in the deletion of ATG in exon 2 (Fig. 2e). The ATG in exon 3 was thus considered to function as the start codon (Fig. 2f).

This patient had typical features of CS syndrome, and three probably pathogenic variants were detected on *ERCC6*. Two of them, c.1931T>C p.(Ile644Thr) and c.1936del p.(Asp646Thrfs*52), were inherited from the mother, indicating that both were on the same allele. The c.1627A>T (p. Ile543Phe) variant might have been inherited from his father, or it might be a de novo mutation (Fig. 1a).

Through genetic analysis via whole-exome analysis of this patient without the ATR-XS phenotype, we also detected the variant c.21-1G>A in *ATRX* (Fig. 1a). We confirmed that exon 2 was spliced out of the patient’s mRNA, causing early truncation of ATRX (Fig. 2e). CS patients also have ID and a particular facial phenotype. Thus, the contribution of the *ATRX* mutation to ID in this patient could not be evaluated, and the facial phenotype might have been masked. However, the loss of genital anomalies and alpha thalassemia indicated that the contribution of the *ATRX* variant to this patient was minor. ATRX expression was confirmed on the patient’s cells by an antibody to the C-terminal region of ATRX (Fig. 2h, i). The possibility that the normally spliced product might have been produced somehow was unlikely, as RT‒PCR detected only the product without exon 2. Another explanation was that translation might have started from the altered start codon (Fig. 2f). In the databases, including GenBank, proteins translated from ATG in exon 3 were reported. Their protein IDs were XP_016885097.1, XP_016885100.1, XP_006724730.1, XP_016885092.1 and XP_005262213.2. These proteins had almost the entire ATRX sequence and lacked only the first 33 of 2492 amino acids. Although these were proposed from automated computational predictions, some of these alternately initiated proteins were considered to be translated and functioned to prevent ATR-XS in our patient. Chudley–Lowry syndrome (ChLS, MIM 309490)^10^ is characterized by ID, short stature, hypogonadism and distinctive facial features of a depressed nasal bridge, anteverted nares, inverted-V-shaped upper lip and macrostomia^10^. In a patient with ChLS, an R37X variant on exon 2 of *ATRX* was reported. ATRX was expressed in the patient’s lymphoblastoid cells, and three types of alternative splicing from exons 1 to 3 of *ATRX* using different initiation codons were detected. One of the alternately spliced products did not have exon 2 from the alternative splicing donor site of exons 1 to 3 and escaped early termination. In this patient, the amount of functional ATRX protein might be lower because certain transcripts that could not produce working proteins existed. ChLS was considered to be allelic to ATR-XS, with its less severe phenotype being due to the presence of a certain level of ATRX proteins^10^. In our patient, it was considered that only the transcript without exon 2 had been transcribed, with the full amount of functional ATRX translated.

Because the *ATRX* variant in this patient was detected coincidentally, it is possible that variants in exons 1 to 3 existed without the phenotype and could be detected by chance on an exome analysis for other diseases. Genetic counseling is important when such variants are detected to judge whether the mutation is pathogenic.

## HGV database

The relevant data from this Data Report are hosted at the Human Genome Variation Database at 10.6084/m9.figshare.hgv.3229. 10.6084/m9.figshare.hgv.3232. 10.6084/m9.figshare.hgv.3235. 10.6084/m9.figshare.hgv.3238.

## Supplementary information

Supplemental figure
