# Supplementary figures and images for: The ATRX splicing variant c.21-1G>A is asymptomatic
Source: Hum Genome Var. 2022 Sep 14;9:33. doi: 10.1038/s41439-022-00212-x (PMC9474544; doi:10.1038/s41439-022-00212-x)

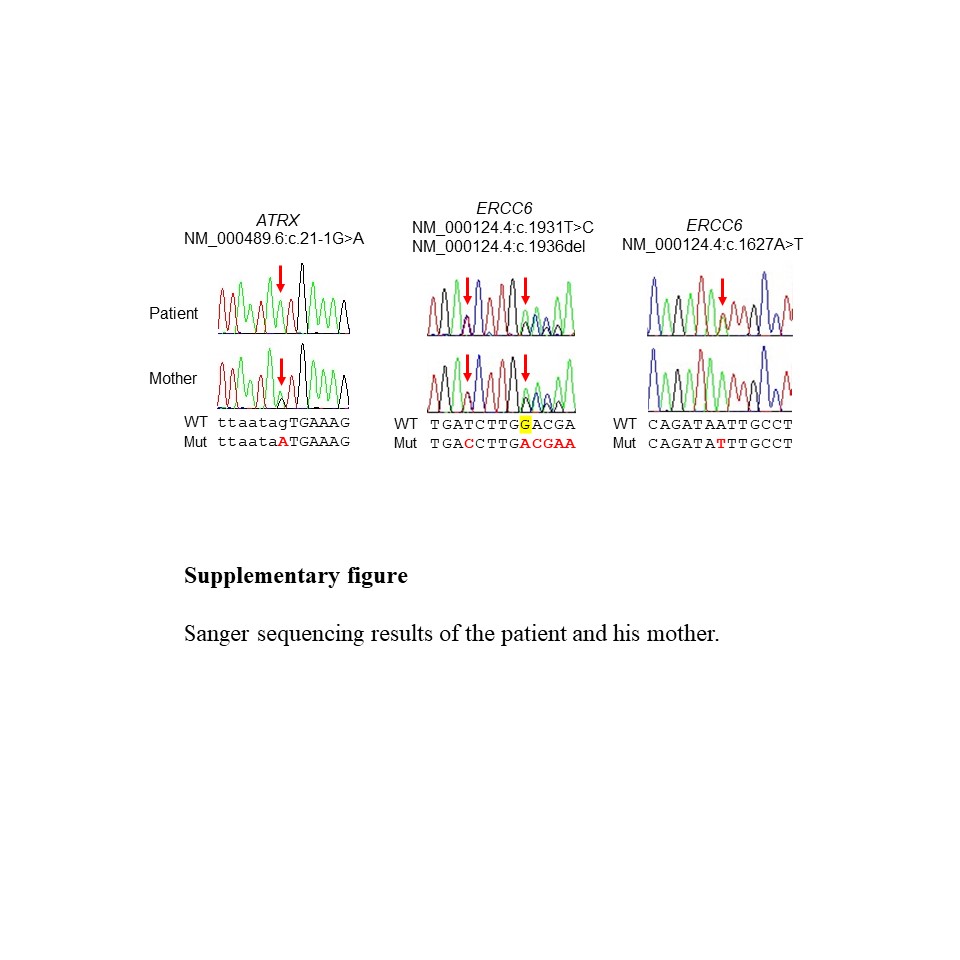

Supplement: Supplementary file 1 — Supplemental figure [file 41439_2022_212_MOESM1_ESM.jpg]
